# Biodegradable poly (lactic acid-co-glycolic acid) scaffolds as carriers for genetically-modified fibroblasts

**DOI:** 10.1371/journal.pone.0174860

**Published:** 2017-04-05

**Authors:** Tatjana Perisic, Ziyang Zhang, Peter Foehr, Ursula Hopfner, Kathrin Klutz, Rainer H. Burgkart, Alexei Slobodianski, Moritz Goeldner, Hans-Günther Machens, Arndt F. Schilling

**Affiliations:** 1 Experimental Plastic Surgery, Department for Plastic Surgery and Hand Surgery, Klinikum rechts der Isar, Klinikum rechts der Isar, Technische Universität München, Munich, Germany; 2 Department of Orthopedics, Tongji Hospital, Tongji Medical College, Huazhong University of Science and Technology, Wuhan, Hubei, China; 3 Department of Orthopaedics and Sportsorthopaedics, Klinikum rechts der Isar, Technische Universität München, Munich, Germany; 4 Johnson & Johnson MEDICAL GmbH, Norderstedt, Germany; 5 Klinik für Unfallchirurgie, Orthopädie und Plastische Chirurgie, University Medical Center Göttingen, Göttingen, Germany; Helsingin Yliopisto, FINLAND

## Abstract

Recent advances in gene delivery into cells allow improved therapeutic effects in gene therapy trials. To increase the bioavailability of applied cells, it is of great interest that transfected cells remain at the application site and systemic spread is minimized. In this study, we tested clinically used biodegradable poly(lactic acid-co-glycolic acid) (PLGA) scaffolds (Vicryl & Ethisorb) as transient carriers for genetically modified cells. To this aim, we used human fibroblasts and examined attachment and proliferation of untransfected cells on the scaffolds *in vitro*, as well as the mechanical properties of the scaffolds at four time points (1, 3, 6 and 9 days) of cultivation. Furthermore, the adherence of cells transfected with green fluorescent protein (GFP) and vascular endothelial growth factor (VEGF165) and also VEGF165 protein secretion were investigated. Our results show that human fibroblasts adhere on both types of PLGA scaffolds. However, proliferation and transgene expression capacity were higher on Ethisorb scaffolds most probably due to a different architecture of the scaffold. Additionally, cultivation of the cells on the scaffolds did not alter their biomechanical properties. The results of this investigation could be potentially exploited in therapeutic regiments with areal delivery of transiently transfected cells and may open the way for a variety of applications of cell-based gene therapy, tissue engineering and regenerative medicine.

## Introduction

New generation non-viral gene delivery systems, such as nanoparticles, novel cationic lipids and polymers, chemically coupling the nucleic acid to peptides and polymers [1–9] have shown to overcome some of the limitations of viral gene transfer and offer advantages in use of recombinant proteins [10, 11]. On the other side, the localized delivery of genetically modified cells themselves poses another major barrier for efficient and area-specific therapeutic protein expression in gene therapy applications. Challenges regarding the reduction of off-target effects remain to be addressed [12]. Thus, biodegradable synthetic polymer scaffolds may offer a safe and effective option for targeted cell delivery.

Biodegradable scaffolds are routinely used as reinforcement material for various surgical procedures, including hernia repair [13], tendon reconstruction [14], cranio-maxillo-facial surgery or neurosurgery [15–18]. Synthetic polymers such as polylactic acid (PLA), polyglycolic acid (PGA) and their copolymer poly(lactic acid-co-glycolic acid) (PLGA) are also highly useful for the temporary management of pathologically altered tissue architectures including ligaments, skin, vascular tissues and skeletal muscle [19]. The implanted scaffolds not only provide structural support but also guide new tissue ingrowth and, importantly, are completely absorbed by the body without the need for subsequent surgical removal. As an alternative to fully synthetic polymer-based scaffolds, decellularized biological matrix material of xenogenic or allogenic origin can be used [20]. Biological scaffolds exhibit a tensile strength comparable to that of PLGA-based scaffolds and show superior collagen deposition and organisation [21]. Still, xenografts and allografts have serious constrains due to limited availability, residual α-Gal-mediated immunogenicity [22], as well as the risk of transmission of animal- or human-derived infectious agents [23]. Biodegradability of the matrix material is a crucial issue in the development of tissue engineering scaffold structures. By utilizing easy manufactured off-the-shelf synthetic absorbable polymers, complications associated with antigenicity and disease transmission can be eliminated.

Another important factor in scaffold-aided tissue regeneration is that this process is critically dependant on efficient vascularization [24–28]. Moreover, local delivery of recombinant angiogenic growth factors from scaffold materials presents a promising strategy in promoting formation of a functional blood vessel network within the regenerated tissue [29, 30]. However, the short half-life of the recombinant proteins hinders the unleashing of their full angiogenic potential. Given the essential role in reparation of damaged tissue, fibroblasts are particularly suitable as cellular vehicles for delivery of angiogenic growth factors in tissue engineering [31] as well as for gene therapy applications [32]. Thus, in this study, we applied an expression vector for vascular endothelial growth factor (VEGF165) as a model with which we studied the utility of PLGA-based scaffolds as carriers for genetically modified cells. We used an established transfection procedure on human Hs27 fibroblasts and tested their proliferation and morphology on two PLGA-based scaffolds Vicryl and Ethisorb. The rationale behind using these scaffolds is that they are already established in clinical use and PLGA has been shown to provide excellent conditions for attachment, growth and motility of human skin fibroblasts [33]. Furthermore, we investigated mechanical properties of the scaffolds seeded with cells over the short time frame of 9 days. Finally, Vicryl and Ethisorb were compared in terms of their ability to serve as adhesion stratums for VEGF165-overexpressing Hs27 cells and production of angiogenic VEGF165 protein.

## Materials and methods

### Cells and Vicryl and Ethisorb scaffolds

Human fibroblast cell line Hs27 (ATCC; CRL-1634) was cultured under standard condition (37°C, 5% CO_2_) in Dulbecco's Modified Eagle Medium (DMEM) containing phenol red, stable glutamine and 4 g glucose/l (PAA, Coelbe, Germany). The culture medium was supplemented with 10% fetal calf serum (FCS: heat inactivated FCS-Gold, PAA, Coelbe, Germany) and antibiotic/antimycotic solution (AB/AM; PAA, Coelbe, Germany). DMEM containing FCS and AB/AM solution is referred herein as growth medium. For this study, two types of absorbable surgical scaffolds were used: Vicryl (VM 802) and Ethisorb (Patch Typ 6; ZVP 203) scaffold (Ethicon, Johnson & Johnson Medical, GmbH, Norderstedt, Germany). The Vicryl scaffold consists of polyglactin 910, which is a co-polymer of 90% glycolide and 10% L-lactide. Ethisorb is also a polyglactin 910-based implantable scaffold and it consists mainly of polyglactin 910 and a small proportion of poly-P-dioxanon. The resorption time for polyglactin 910 is 45–60 days and for poly-P-dioxanon 90–180 days [34].

### Cell growth on Vicryl and Ethisorb scaffolds

For both types of scaffolds, a cell suspension of 5 x10^5^ Hs27 cells in 4 ml growth medium was pipetted in 4-well rectangle plates (Thermo Fisher Scientific, Waltham, MA, USA). Vicryl and Ethisorb patches were immediately placed inside. The scaffolds were incubated overnight in a cell incubator under standard conditions on a horizontal shaker (Duomax 1030,Heidolph, Germany). For controls, the scaffold was put in 4 ml growth medium without Hs27 cells and handled the same way as the scaffold seeded with human fibroblast cells. To remove Hs27 cells which did not attach to the scaffold, 16–24 hours after initial seeding, the scaffold with adherent cells was transferred in new 4-well rectangle plates filled with 4 ml fresh growth medium per well and further incubated under standard conditions. Proliferation rate, morphology, and distribution of attached Hs27 cells, as well as the mechanical properties of the scaffold populated with cells were examined 1, 3, 6 and 9 days after the seeding of cells.

### Measurement of Hs27 cell proliferation activity on Vicryl and Ethisorb scaffolds

Cell proliferation activity was measured by ready-to-use colorimetric WST assay (Roche Molecular Biochemicals, Basel, Switzerland). For that, all specimens were transferred in a new rectangle 4-well plate with 3 ml fresh growth medium supplemented with WST solution and incubated for 2 hours at 37°C. The assay principle is based upon the reduction of the tetrazolium salt WST to formazan by cellular dehydrogenases. The generation of the dark yellow coloured formazan is measured at 450 nm and is directly correlated to cell number. The measurement was performed according to the manufacturer’s instructions by using a microplate reader (Mithras LB 940, Berthold Technologies GmbH, Germany).

### Visualisation of morphology of Hs27 cells grown on Vicryl and Ethisorb scaffolds

In order to investigate the cell morphology, scaffolds seeded with Hs27 cells were fixed either in 3% glutaraldehyde or in 3,7% formaldehyde. Two different methods for cell visualisation were applied: Giemsa staining and scanning electron microscopy (SEM). For Giemsa staining, formaldehyde-fixed specimens were stained for 10 min in a 1:20 pre-diluted Giemsa-solution (PAA, Coelbe, Germany) and rinsed 3 times in Ultra pure water. Photos were taken with a CCD-camera under the microscope (Nikon; Eclipse TE2000-S). For SEM, glutaraldehyd-fixed scaffolds were dehydrated with graded ethanol, dried and sputtered with gold (Baltec SCD005; 40 mA; 80 s). Control scaffolds were handled the same way as cell-seeded scaffolds. SEM was carried out using a Jeol-SEM-5400 (Eching) in Hi-Vac mode by applying an acceleration voltage of 5 kV and detecting secondary electrons for imaging.

### Measurement of mechanical properties of cell-seeded and control Vicryl and Ethisorb scaffolds

The mechanical properties of Vicryl and Ethisorb scaffolds were tested during tensile testing. A universal test system (zwicki1120, Zwick/Roell, Ulm, Germany) was used, equipped with a 2.5 kN load cell (Type KAF-Z class 0.1, A.S.T., Dresden, Germany). The samples were clamped along their longer side in axial direction. To avoid shear stresses and torsional moments a passive x-y-M_z_-compensation tool was inserted between the upper clamp and the load cell. A free length of L_0_ = 20 mm was defined as an elongation of 0%. Subsequently, the sample was stretched at a constant speed of 10 mm/min until total failure of the sample. Tensile testing was stopped automatically by the test system after a force drop of 40% occurred, with respect to maximum force. Maximum force was determined from the highest force value of the force-displacement graph. All mechanical results were summarized as mean values (mean) and standard error of the mean (SEM).

### Transfection of Hs27 cells with GFP and VEGF165 plasmids and cultivation on Vicryl and Ethisorb scaffolds

For transfection experiments, human fibroblasts were cultivated under standard conditions in growth medium in T175 flasks. Upon reaching confluence, 5 x 10^6^ cells were transfected with 20 μg plasmid DNA coding for green fluorescent protein (GFP) provided in the fibroblast transfection kit (Lonza, Cologne, Germany). The transfection was performed by using Nucleofector transfection apparatus (Lonza, Cologne, Germany) and DT130 transfection program. After 16 hours cultivation, the cells were trypsinized, washed and transferred into tubes, which contained pre-cutted Vicryl or Ethisorb scaffolds (1,5 cm x 1,5 cm). The tubes were gently rotated in an incubator for additional 16 hours. Subsequently, the scaffolds were taken out and carefully laid in another plate with fresh growth medium and further cultivated for a defined period (1, 3, 6 and 9 days) under standard conditions. By using the same protocol, human fibroblasts were transfected with 20 μg VEGF165 plasmid DNA [32, 35] and grown on Vicryl or Ethisorb scaffolds. After 1, 3, 6 and 9 days, the supernatant was collected, temporarily stored at -20°C and used for subsequent measurements of VEGF165 protein concentration.

### Detection of intracellular GFP expression and extracellular VEGF165 protein levels

The temporal expression of GFP protein in Hs27 cells was visualised by using the fluorescent microscope (Zeiss Axio Observer.A1, Software AxioVision). The level of VEGF165 protein secreted from Hs27 cells grown on Vicryl and Ethisorb scaffolds was determined by using Human VEGF Quantikine ELISA Kit (R&D Systems) according to the manufacturer's instructions. The absorbance at 450 nm was measured with a microplate reader (Mithras LB 940, Berthold Technologies GmbH, Germany).

### Statistical analysis

The data for the proliferation activity and tensile testing are from single experiments with five technical replicates. The statistical comparison was performed by two-way ANOVA with Tuckey's post-hoc testing. ELISA experiment was performed three times with three technical replicates. The statistical testing was done by two-way ANOVA followed by Sidak’s multiple comparison tests. For all statistical tests, differences among means were considered significant when the p value was < 0.05.

## Results

### Proliferation of Hs27 cells on Vicryl and Ethisorb scaffolds

**Fig 1:**
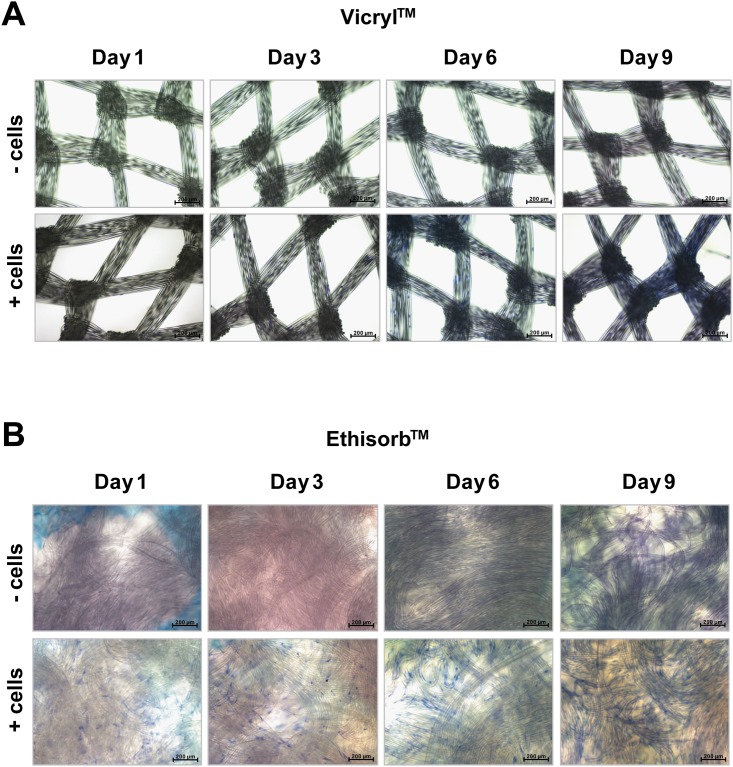
Visualisation of Hs27 cell growth on Vicryl and Ethisorb scaffolds. During the co-cultivation period of 9 days, Hs27 fibroblast cells on Vicryl (A) and Ethisorb (B) scaffolds were investigated at four different time points by means of light microscopy with 100-fold magnification. In the upper row of each panel, the scaffolds containing no cells (control) are presented. The fibroblasts were visualized with Giemsa staining and can be seen in the images of the bottom row of each panel as small blue dots.

**Fig 2:**
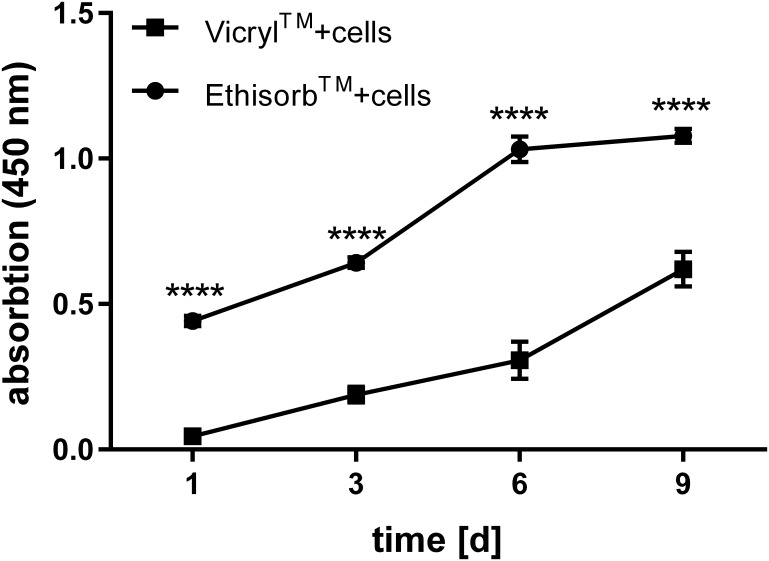
Comparison of proliferation activity of Hs27 cells grown on Vicryl and Ethisorb scaffolds. Hs27 cells were seeded on Vicryl and Ethisorb scaffolds and cultivated for a period of 9 days. At day (d) 1, 3, 6 and 9 the proliferation activity was measured by means of colorimetric WST assay. The results of one experiment are presented as means of absorbance at 450 nm. The error bars represent SEM of five technical replicates (n = 5; two-way ANOVA comparing proliferation activity of cells between Vicryl and Ethisorb for each time point: ****p<0.00005).

Hs27 cell proliferation could be observed on images of cell-seeded Vicryl and Ethisorb scaffolds captured with the light microscope in the course of 9 days (Fig 1). At day one, the cells were scarcely distributed on both types of scaffolds. After 9 days of cultivation, most of the surface of both scaffolds was covered with Hs27 cells. Furthermore, on the images obtained by light microscopy, it is visible that the textile structure of Vicryl scaffold is woven and contains small pores whereas that of Ethisorb is nonwoven. This implicates the difference in the surface area between Ethisorb and Vicryl scaffolds. In order to quantify the changes in cell number observed by the light microscope, the WST assay was applied. As seen on Fig 2, the increase in cell number is reflected by an increase in dehydrogenase activity over the time course of the experiment. When compared to Vicryl scaffolds, the Hs27 cells on Ethisorb scaffolds showed higher values at all time points, as measured by an increase in optical density of formazan dye at 450 nm, probably caused by the increased surface area available to the cells. The parallel increase of the absorbance in both groups over time reflects a similar rate of proliferation of the attached cells on both scaffolds.

### Morphology of Hs27 cells on Vicryl and Ethisorb scaffolds

**Fig 3:**
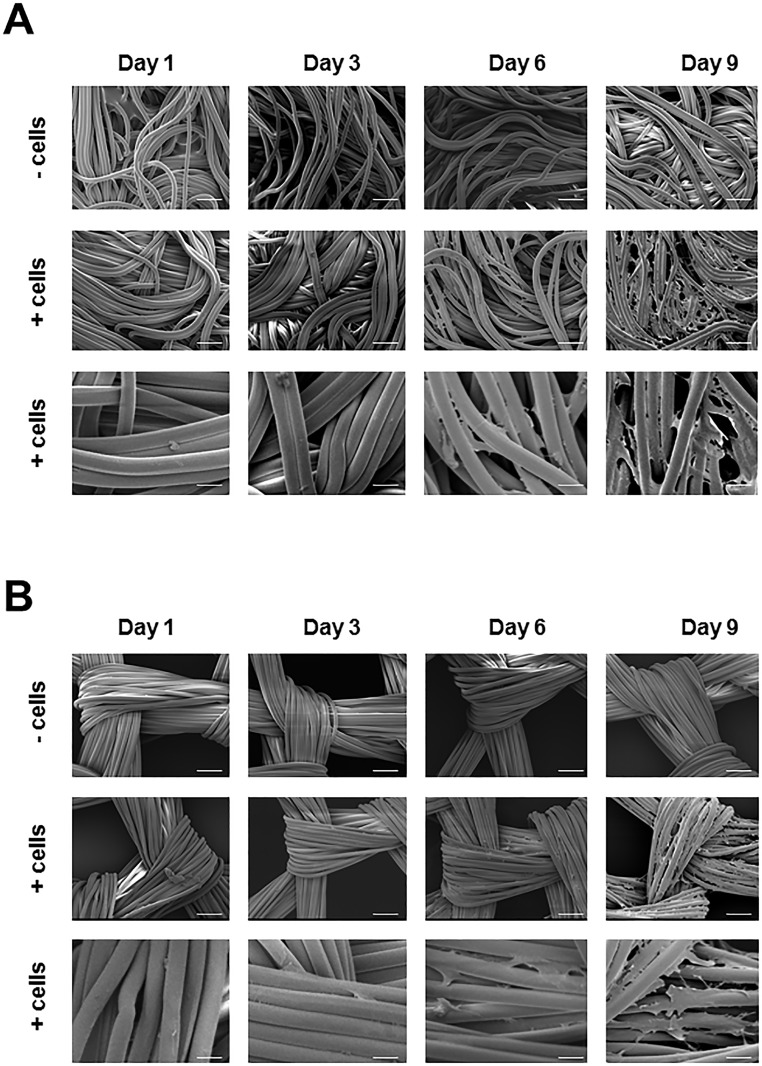
Analysis of scaffold fibers and Hs27 cell morphology by scanning electron microscopy. Hs27 fibroblasts are grown on Vicryl and Ethisorb scaffolds and visualized at day 1, day 3 day6 and day 9 in culture by means of scanning electron microscopy (Scale bars in the upon two rows in A and B are 100μm, in the lowest row are 25μm)

A suitable way to investigate the topography and morphology of rough surfaces is SEM. In this study, it was used for a close inspection of morphology of adherent cells and the physical state of the scaffold fibers. The SEM did not show any obvious signs of degradation of the polymer fibers of Vicryl and Ethisorb scaffolds (with or without attached Hs27 cells) after 9 days in culture (Fig 3). Furthermore, there was a nearly confluent layer of cells visible on both scaffolds at day 9. The cells grown on scaffolds have maintained their typical polygonal flattened shape. The SEM also demonstrated that the attached cells are predominantly located between the polymer fibers of the scaffolds, bridging the inter-fiber gaps with multiple cell protrusions.

### Mechanical properties of Vicryl and Ethisorb scaffolds populated with and without Hs27 cells

**Fig 4:**
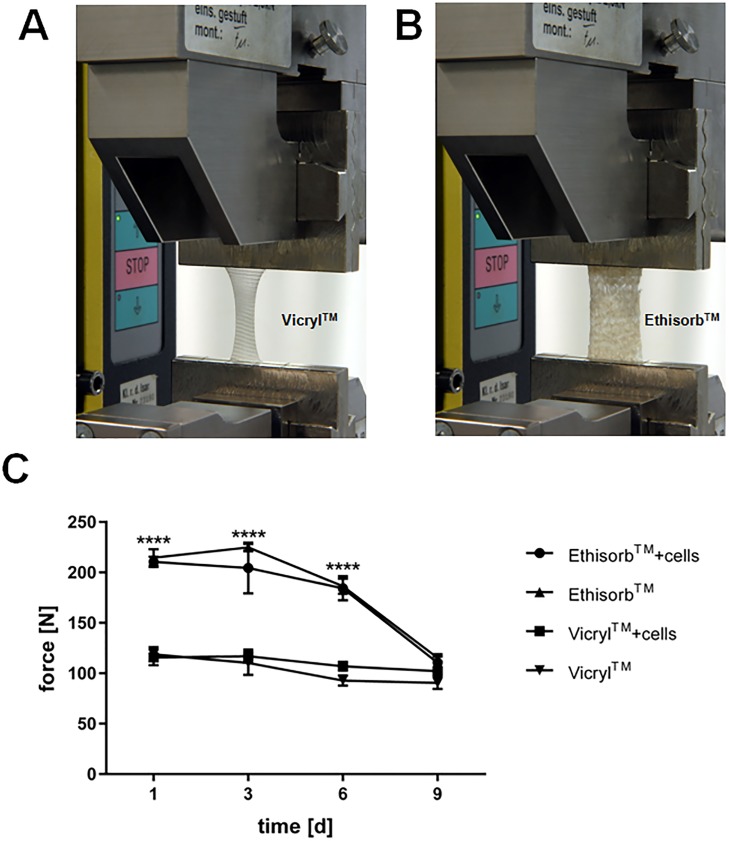
Determination of mechanical properties for Vicryl and Ethisorb scaffolds with and without seeded cells. The mechanical properties of Vicryl (A) and Ethisorb (B) scaffolds were measured by using a uniaxial test system. Meshes were clamped along their long side with an initial length L_0_ of 20 mm. Maximum force values for the two types of scaffolds (both with and without Hs27 cells) were measured in a single experiment at day (d) 1, 3, 6 and 9 and presented graphically (C). All error bars attached to the mean values represent the SEM of five technical replicates (n = 5; two-way ANOVA comparing maximum force values of Vicryl and Ethisorb scaffolds for each time point: ****p<0.00005).

Next, the mechanical strength of Vicryl and Ethisorb scaffolds (with and without attached Hs27 cells) was investigated using tensile testing (Fig 4A and 4B).

The presence of cells did not affect the mechanical behaviour of the scaffolds for both Vicryl and Ethisorb at any time points investigated (Fig 4C).

Furthermore, the mean values of the maximal tensile force for Vicryl scaffolds (with and without cells) only slightly decreased over the course of the 9 days. On the other side, the maximal tensile force of the Ethisorb scaffolds (with and without cells) dropped by almost 50% at day 9. Under the used experimental conditions, Ethisorb scaffolds had significantly higher tensile strength at days 1, 3 and 6 compared to Vicryl.

### Transient GFP and VEGF165 expression in Hs27 cells grown on Vicryl and Ethisorb scaffolds

**Fig 5:**
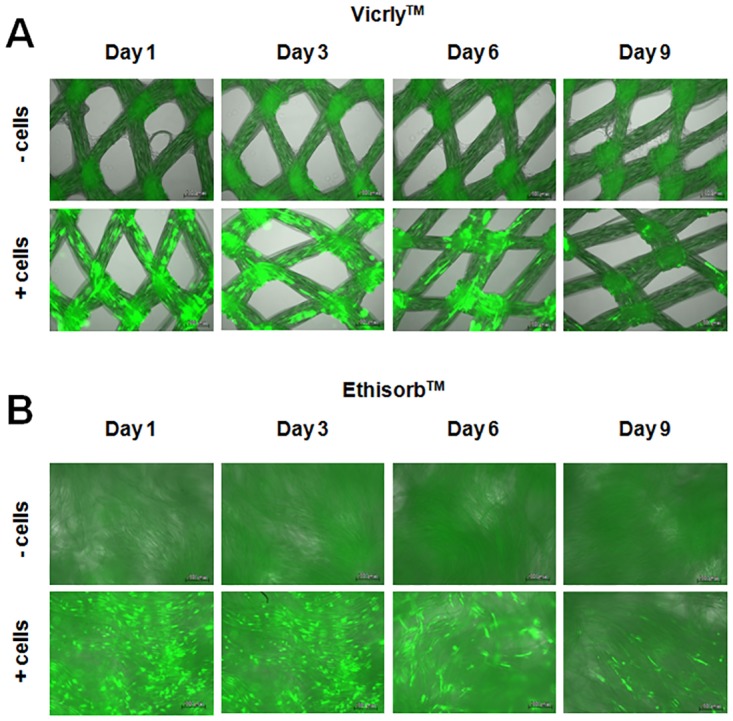
Visualisation of GFP-expressing Hs27 cells adhered on Vicryl and Ethisorb scaffolds. 5 x 10^6^ Hs27 cells were electroporated with 20 μg GFP plasmid by using Nucleofector transfection apparatus. After a short period in culture, transfected cells were transferred on scaffolds. The expression of GPF protein in Hs27 fibroblast grown on Vicryl (A) and Ethisorb (B) scaffolds was recorded at day 1, 3, 6 and 9 by a fluorescent microscope with 100-fold magnification. In the upper row of each panel, the scaffolds containing no cells are depicted.

**Fig 6:**
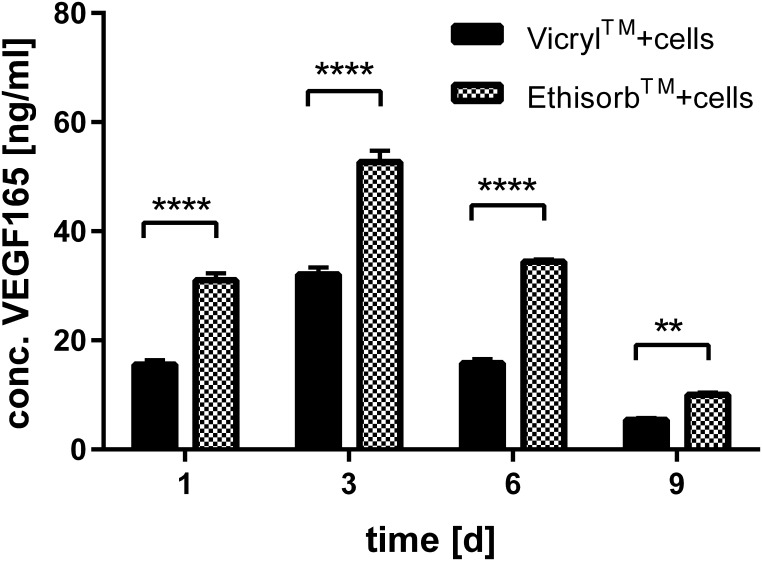
Comparison of VEGF165 protein secretion from Hs27 cells grown on Vicryl and Ethisorb scaffolds. Human fibroblasts were transfected with 20 μg VEGF165 plasmid DNA and co-cultured with Vicryl or Ethisorb scaffolds. After 1, 3, 6 and 9 days (d) the supernatant was collected and the level of VEGF165 protein was determined by the enzyme-linked immunosorbent assay. Data shown are mean ± SEM from three experiments with three technical replicates (n = 3; two-way ANOVA comparing VEGF165 concentration of cells grown on Vicryl and Ethisorb scaffolds for each time point: **p<0.005, ****p<0.00005).

Finally, the difference in the level of GFP expressed in Hs27 cells and the VEGF165 protein secreted from Hs27 cells grown on Vicryl and Ethisorb scaffolds was investigated. By using the GFP expressing plasmid a general transfectability of the Hs27 cells was tested and whether they adhere on the scaffolds upon transfection. Both GFP and VEGF165 transfected cells successfully adhered on the surface of the scaffolds (Fig 5A and 5B). For both Vicryl and Ethisorb, the expression of GFP protein was the highest on day 1 and day 3 and then gradually decreased on day 6 and day 9 Similarly, the peak of VEGF165 protein expression from cells grown on Vicryl and Ethisorb scaffolds was reached on day 3 with gradual decrease in the course of next 6 days (Fig 6). At all investigated time points, the level of VEGF165 protein secretion from Hs27 cells grown on Ethisorb scaffolds was significantly higher than the level of VEGF165 protein secretion from cells grown on Vicryl scaffolds.

## Discussion

Due to significant advances in recombinant DNA technology, cell-based gene therapy is increasingly recognized as an ideal tool to deliver therapeutic proteins to target sites. A typical way of administering genetically modified cells is the direct injection of cells in target areas. However, this mode of administration is accompanied by the spread away from the injection site. The spreading of transfected cells can reduce vector levels at the target site and also lead to low-grade systemic spread in off-target regions [35]. There are different strategies in addressing those concerns. For example, in adoptive T cell therapy, new generation of chimeric antigen receptors called TRUCKs (T cells redirected for universal cytokine killing) are used as vehicles to produce and release a transgenic product that accumulates in the targeted tissue [36] and this strategy can potentially yield effective and localized therapeutic effects. Another approach in targeted cancer gene therapy of solid tissues is based on local delivery of NK4 (hepatocyte growth factor antagonist) secreted from an NK4 gene-transduced oral mucosal epithelial cell [37]. A similar technique by using cellular vehicle for delivery of bone morphogenetic protein-2 has been used to promote bone regeneration [38]. Thus, genetically transfected cells can be used for delivery of therapeutic cells to the intended area in the body.

In general, growing living cells on scaffolds for tissue engineering purposes has been shown to offer significant clinical benefits [39, 40]. The cell-material interaction is very complex and seeded cells respond differently to specific surface chemistry and various architectural parameters [41]. In particular, the choice of scaffold material determines cell adhesion, which has direct effects on cell behaviour including morphology and proliferation. Unchanged cellular phenotype is an important aspect of biocompatibility. The cells show scaffold preferences and, thus, the suitability of the material must be validated beforehand [42, 43]. The results of our study indicate that Hs27 cells grow well on both investigated surgical scaffolds and show apparently unchanged and typical fibroblast morphology. As for the proliferation rate, our results demonstrate that attached Hs27 cells proliferate healthily on both investigated materials. This is indicated by microscopical evaluation as well as by quantification of the metabolic rate of the cells. To measure metabolism, we used a WST-1 test. Similar to the MTT method, the WST-1 system is based on the conversion of tetrazolium to formazan. Another frequently used system to study cell metabolism is based on the risazurin/resorufin conversion (e.g. Alamar blue, CellTiterblue). However, as these systems measure fluorescence instead of absorbance they tend to be influenced by fluorescent parts of the studied system and therefore we preferred WST-1 in this case [44]. In our study, although we started with the same amount of cells on all scaffolds, there were eventually more cells on Ethisorb scaffolds than on Vicryl. In contrast to Vicryl, which has a woven scaffold-structure with small pores, Ethisorb consists of nonwoven fibres resembling a fleece. This implicates that Ethisorb provides a higher surface area compared to Vicryl allowing a greater number of cells to colonize and expand and, thus, likely explains their greater metabolic activity on Ethisorb scaffolds. Another interesting phenomenon we observed was that cells spread on the entire surface of the scaffolds and at the later time points start to grow between the filaments of the respective materials. Due to the manufacturing process of the materials there are relatively large pores (ca. 300μm) between the respective cords of the Vicryl mesh, while the surface of Ethisorb mesh is dense. It seems that this leads to a higher area for cell growth on Ethisorb, which may also explain the higher VEGF expression with the Ethisorb mesh. Considering the restricted availability of autologous fibroblasts for clinical use, the pore size of engineered scaffolds could therefore pose one of the limiting factors for gene therapy-based soft tissue reconstructions.

Apart from the influence of the scaffold material on growth, proliferation and differentiation of seeded cells, mechanical and degradation properties of biodegradable scaffolds may be affected by the cells [16, 45–48]. Surgical scaffold materials and tissue engineering constructs must, first of all, provide sufficient mechanical support at the defect site. Ideally, the mechanical properties of the scaffold material should not be affected by the cells which are, in a pre-implantation phase, grown on their surface. Therefore, in this study, we tested whether Hs27 cells influence the mechanical behaviour of Vicryl and Ethisorb scaffolds in the course of the experiment. Of note, under our experimental conditions, fibroblasts were cultured as a monolayer without adding extracellular matrix components. The results presented here show that attached cells did neither lead to additional deterioration of the used scaffolds after 9 days nor to protection of the fibers from hydrolytic degradation. Interestingly, Ethisorb lost mechanical resistance much faster than Vicryl, although its components (much smaller pore size) should theoretically degrade slower than the components of Vicryl [34].

The ability of cell therapy using genetically modified cells to deliver a bioactive protein at the target site circumvents the limitations associated with conventional protein delivery strategies. In this study, we made use of a well-established transfection method to generate VEGF165-overexpressing fibroblast, which colonized efficiently the surface of Vicryl and Ethisorb scaffolds. VEGF is a well-established therapeutic protein for enhancing angiogenesis both *in vitro* and *in vivo*. Our and many other groups have previously shown that secreted VEGF can enhance cell proliferation, wound healing and angiogenesis in different pre-clinical models [24, 49, 50]. In present study, our main focus was not on the effect of *VEGF* on proliferation of cells on scaffolds but rather if scaffolds can be used as carriers for cells which are genetically engineered to secrete VEGF or other therapeutic proteins. The VEGF165 protein expression profiles were similar between the investigated scaffolds showing a gradual decrease in protein levels. The Hs27 cells grown on Ethisorb expressed higher levels of VEGF165 protein, probably due to greater surface-to-volume ratio of Ethisorb scaffolds and higher number of attached cells. In this study, Hs27 were transiently transfected with a VEGF165 expression plasmid. This short-termed expression of VEGF165 protein is thought to stimulate biomaterial vascularisation without negative consequences of prolonged angiogenic stimulation. Our results show that the level of VEGF165 expression of Hs27 cells grown on scaffolds reaches the therapeutic levels as demonstrated in the ischemic hind limb animal model [32]. There are concerns about a possible oncogenic potential of genetically modified cells. These are mainly based on applications with stable viral transfection, because this can induce oncogenic mutations through random integration [51]. To avoid this problem, we have used here a plasmid vector approach, which has an excellent safety profile, because the plasmid is not expected to be integrated into the host genome and therefore the risk is much lower.

Although these *in vitro* findings present an important step toward construction of bioactive PLGA-based gene delivery cell carriers, further investigations with different therapeutic proteins are needed to determine their clinical utility. Moreover, although it is important to test the in vitro methods *in vivo*, we think it is not necessary to perform the *in vivo* study for the current *in vitro* method. First, several former studies including ours have already proven the use of VEGF as a therapeutic protein for improving angiogenesis and wound healing both *in vitro* and *in vivo* [24, 48, 49]. Moreover, we have published a pre-clinical study regarding the genetic-modified fibroblasts expression angiogenic factors including VEGF. In this work we actually applied the same transfection method with fibroblasts and VEGF plasmid as in the current one. We found that genetically modified fibroblasts can enhance angiogenesis and arteriogenesis in a hindlimb ischemia model [32]. This study was a proof-of-concept for our transfection method and *in vivo* application. Also, both PLGA-based meshes used in our study are clinical level products used for decades. Thus, our current study is mainly focused on the evaluation of a possible carrier material for such applications. We show here that the cells not only are able to survive and proliferate on the scaffolds but more importantly also produce the desired protein.

In summary, in the present study, we show that human fibroblasts seeded on biodegradable Vicryl and Ethisorb scaffolds show excellent biocompatibility. Furthermore, this model system allows successful genetic modification of the cells. The presented methodology could be easily adapted for other proteins and growth factors allowing a broader use of this gene-enhanced engineering technology.

## Conclusions

Bioresorbable PLGA scaffolds can be used as vehicle for the delivery of transiently transfected cells and may open the way for a variety of applications of gene therapy, tissue engineering and regenerative medicine. Scaffolds with a condensed structure and smaller pore size might lead to a better cell-scaffold interaction and thus lead to a higher yield of the desired recombinant therapeutic proteins.

## Supporting information

S1 Data Raw data of mechanical property test. (XLSX)

S2 Data Raw data of cell proliferation test with WST-1. (XLSX)
